# Diversity and gender at the largest European university hospital: The effects of discrimination on mental health

**DOI:** 10.1192/j.eurpsy.2024.276

**Published:** 2024-08-27

**Authors:** I. Enarovic, V. M. Straßburger, M. Berger, J. Smith, G. Stadler, P. J. Buspavanich

**Affiliations:** ^1^Charité-Universitätsmedizin, Berlin; ^2^Medical School, Hamburg, Germany; ^3^University of Aberdeen, Aberdeen, United Kingdom; ^4^Brandenburg Medical School Theodor Fontane, Neuruppin, Germany

## Abstract

**Introduction:**

Discrimination is known to have different effects on health. In particular the mental health of affected people diminishes. Although it is known that marginalized groups are discriminated against more, at present only research on gender and ethnicity has been done. Further diversity domains like socioeconomic status, care responsibilities, sexual orientation, disability, mental and physical health, and their intersections have been scarcely looked at.

**Objectives:**

The aim of the study was to determine the effects of discrimination on the mental health for employees and students of a university hospital taking diversity domains into account.

**Methods:**

A web-based survey between June 22 to October 23 was conducted using the PHQ-4 and WHO-5 as well as innovative Diversity Minimal Item set to measure different diversity domains.

**Results:**

Preliminary data shows that discrimination among employees and students is common, widespread and most frequent based on gender, ethnicity and health. The mental health of those who feel discriminated against tends to be poorer, especially looking at the intersectionality of diversity domains.

**Conclusions:**

The results of this study suggest that both more measures to prevent discrimination in a university hospital have to be implemented and individuals from marginalized groups need special psychosocial support to ensure a safer working environment. In addition, greater attention to diversity and inclusion in medical research is needed to develop appropriate responses and interventions, including diversity policies.

**Disclosure of Interest:**

None Declared

